# Phenotypic plasticity under rapid global changes: The intrinsic force for future seagrasses survival

**DOI:** 10.1111/eva.13212

**Published:** 2021-04-04

**Authors:** Jessica Pazzaglia, Thorsten B. H. Reusch, Antonio Terlizzi, Lázaro Marín‐Guirao, Gabriele Procaccini

**Affiliations:** ^1^ Department of Integrative Marine Ecology Stazione Zoologica Anton Dohrn Naples Italy; ^2^ Department of Life Sciences University of Trieste Trieste Italy; ^3^ Marine Evolutionary Ecology GEOMAR Helmholtz Centre for Ocean Research Kiel Kiel Germany; ^4^ Department of Biology and Evolution of Marine Organisms Stazione Zoologica Anton Dohrn Naples Italy; ^5^ Seagrass Ecology Group Oceanographic Center of Murcia Spanish Institute of Oceanography Murcia Spain

**Keywords:** acclimation, adaptation, genetic diversity, global changes, phenotypic plasticity, reaction norm, seagrasses

## Abstract

Coastal oceans are particularly affected by rapid and extreme environmental changes with dramatic consequences for the entire ecosystem. Seagrasses are key ecosystem engineering or foundation species supporting diverse and productive ecosystems along the coastline that are particularly susceptible to fast environmental changes. In this context, the analysis of phenotypic plasticity could reveal important insights into seagrasses persistence, as it represents an individual property that allows species’ phenotypes to accommodate and react to fast environmental changes and stress. Many studies have provided different definitions of plasticity and related processes (acclimation and adaptation) resulting in a variety of associated terminology. Here, we review different ways to define phenotypic plasticity with particular reference to seagrass responses to single and multiple stressors. We relate plasticity to the shape of reaction norms, resulting from genotype by environment interactions, and examine its role in the presence of environmental shifts. The potential role of genetic and epigenetic changes in underlying seagrasses plasticity in face of environmental changes is also discussed. Different approaches aimed to assess local acclimation and adaptation in seagrasses are explored, explaining strengths and weaknesses based on the main results obtained from the most recent literature. We conclude that the implemented experimental approaches, whether performed with controlled or field experiments, provide new insights to explore the basis of plasticity in seagrasses. However, an improvement of molecular analysis and the application of multi‐factorial experiments are required to better explore genetic and epigenetic adjustments to rapid environmental shifts. These considerations revealed the potential for selecting the best phenotypes to promote assisted evolution with fundamental implications on restoration and preservation efforts.

## 1 INTRODUCTION

In the context of global environmental changes, studying the ability of species to cope with environmental shifts is fundamental for predicting their fate. The possibility to rapidly respond to environmental changes is exacerbated by the occurrences of different human pressures, which can have critical effects at the ecosystem level, forcing ecological systems into an alternative stable state (Beisner et al., 2003; Harley et al., 2006). The marine coastline is particularly vulnerable to environmental disturbances, such as sea level rise, acidification, increase of temperature, and intensity of heat waves events and storms (IPCC, 2019). Additionally, climate‐derived environmental changes and their consequences on habitats can potentially be intensified by regional anthropogenic pressures, including overfishing and nutrient pollution among others (Chaturvedi et al., 2017; Zaneveld et al., 2016). The resulted exposure to multiple stressors forces coastal marine environments to drastic changes as a consequence of the alteration of species biodiversity, distribution, and ecosystem functioning (Gunderson et al., 2016). Importantly, rapid and extreme environmental changes strongly affect the performance of foundation species (i.e., species with a structural role within an ecosystem) altering the resilience capacity (i.e., the ability to recover and continue functioning after a disturbance) of the entire ecosystem (Thrush et al., 2009). The degree of the ecosystem transition into different states strongly depends upon the foundation species’ tolerance and resistance to environmental variability or disturbances (Scheffer & Carpenter, 2003). These abilities, in turn, rely on different physiological and molecular mechanisms that drive individual or population responses in the presence of relatively rapid environmental changes (Sih et al., 2011; Summers et al., 2012; York et al., 2013). One of the main concerns of rapid shifts is that these changes do not allow species to react swiftly enough in order to cope with and survive in the new more stressful environment. Analyzing how species traits change with the environment becomes thus of crucial importance.

Among higher plants, seagrasses are the only group that has returned to a completely submerged marine life (Shepherd et al., 1989). Although fossil evidence for marine plants is limited, some records indicate that seagrass’ ancestors likely evolved more than 100 Ma ago in the Cretaceous Period, whereas modern seagrass families beginning to diverge more than 70 Ma ago (Hedges & Kumar, 2009). Seagrasses are a polyphyletic group of monocotyledons, belonging to the order of Alismatales which includes 11 families of aquatic–freshwater species and four families that are fully marine (Posidoniaceae, Zosteraceae, Hydrocharitaceae, and Cymodoceaceae; Les et al., 1997). Among the hundreds of thousand species of angiosperms today, there are currently only 12 genera and ca. 65 species of seagrasses (Chase et al., 2016). Seagrasses are widely recognized as key ecosystem engineering or foundation species, supporting diverse and productive ecosystems in the photic zone of the marine coastline around all the continents except Antarctica (Bos et al., 2007). These marine plants fulfill a series of important ecosystem services worldwide, including oxygen production and CO_2_ sequestration (Champenois & Borges, 2019; Duarte & Krause‐Jensen, 2017). Although they occupy only 0.1% of the ocean surface, it is estimated that seagrasses can store 27–44 Tg organic carbon (C_org_) year^−1^ globally, corresponding to the 10–18% of the total carbon stock in the oceans (Fourqurean et al., 2012). As in terrestrial plants, where clonal species are the most abundant members among perennial grasslands (Klimeš et al., 1997), seagrasses are also mostly herbaceous even if stiff and hard stems and rhizomes occur in some families (e.g., Posidoniaceae). A huge variability exists among seagrasses, ranging from species characterized by short‐lived shoots, with a quicker cycle of growth and death of shoots (i.e., Cymodoceaceae), to slow‐growing and long‐lived plants (i.e., Posidoniaceae) (Larkum et al., 2006). Seagrasses often exhibit a mix of sexual and clonal reproduction that has been a crucial aspect of their evolutionary history. Seagrass meadows show high‐genetic variability depending on the interplay between sexual reproduction and clonal growth and by latitudinal and geographical regions (Bricker et al., 2011; Jahnke et al., 2016). As for terrestrial plants, seed dispersal is critical for population distribution, contributing to the maintenance of genetic diversity and the shaping of spatial genetic structure (Kendrick et al., 2012). Theory predicts that the lack of genetic variation leads to the accumulation of deleterious mutations that negatively affect plants’ persistence under environmental shifts (Silvertown, 2008). However, sexual reproduction has a drawback, since it is a costly energetic process that requires resource allocation and depends on surrounding conditions (Diaz‐Almela et al., 2006).

In seagrasses, vegetative (= clonal) reproduction occurs through rhizome extension and branching in space, leading to the formation of extensive underwater meadows (Larkum et al., 2006). The success of clonal propagation is related to different and unique ecological advantages, such as resource and risk sharing, and economies of scale among ramets within a genotype (Dodd & Douhovnikoff, 2016; Ruocco et al., 2020). Thus, clonal plants appear to be more resistant than plants lacking clonal reproduction and are likely more buffered against habitat deterioration (Pennings & Callaway, 2000). This capacity has allowed clonal plants to colonize diverse terrestrial and marine ecosystems, and include many of the most important crops and invasive plants, and some of the earth's largest and oldest plant species (Honnay & Bossuyt, 2005; Pan & Price, 2002).

Seagrass meadows are particularly susceptible to environmental changes. They are exposed to the effects of single and multiple stressors, due to local and global threats, including changes of environmental parameters (i.e., light and salinity levels) and nutrient condition of the water column (Moreno‐Marín et al., 2018; Pereda‐Briones et al., 2019; Salo & Pedersen, 2014). The intensifying destruction of the marine environment is promoting a huge decline of seagrass meadows with knock‐on effects for the entire coastal benthic ecosystem (Boudouresque et al., 2009; Gacia et al., 1999). A complete analysis performed by Waycott et al. (2009) revealed that seagrass loss rates have increased to 7% year^−1^ since 1990, placing seagrasses among the most threatened ecosystems on earth.

How marine clonal plants with low‐genetic recombination have been able to survive to past environmental changes and which are the main implications for current and future environmental shifts are still to be clarified and becomes mandatory for assessing their fate and adopting proactive management actions. An aspect that has to be considered is the longevity that characterizes genets in some species (Arnaud‐Haond et al., 2012; Ruggiero et al., 2002). This points to an intrinsic ability of single genotypes, including mostly clonal populations, to survive and persist across environmental changes (Arnaud‐Haond et al., 2012; Ruggiero et al., 2002). Plastic responses represent an individual property that allow genotypes to accommodate and react to fast environmental changes (Donelson et al., 2019). According to the climate variability hypothesis, seagrass populations living in more dynamic environments and/or at their tolerance limits (e.g., lagoons characterized by unstable salinity or temperature conditions) may better perform in face of environmental changes (Ashander et al., 2016; Botero et al., 2015; Chevin & Hoffmann, 2017; Tomasello et al., 2009). Thus, organisms growing in highly variable environments are more plastic (tolerant) than organisms from more stable environments (Tuya et al., 2019).

**FIGURE 1:**
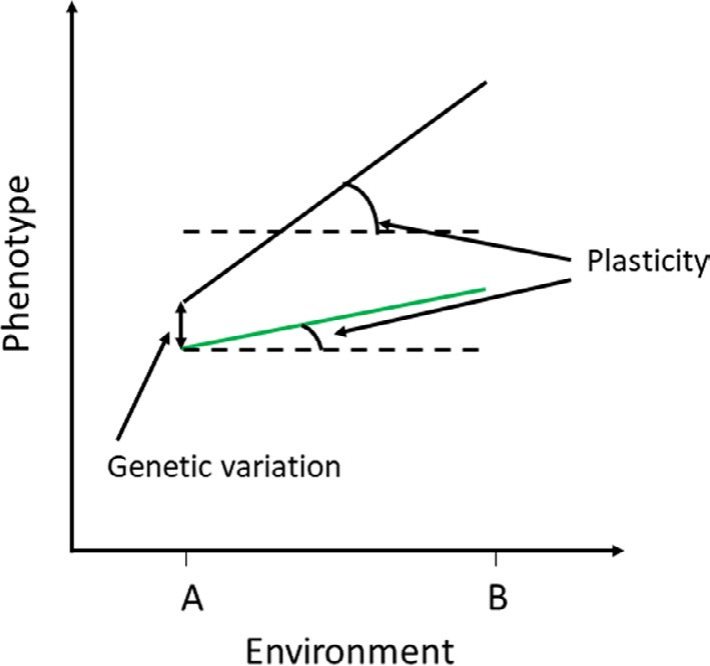
Schematic representation of plasticity resulting from the interaction of linear reaction norms and environments (line slopes). Black and green solid lines refer to different genotypes characterized by genetic variation (line heights); dashed lines refer to the mean phenotypic value across environments (A and B) (from Schlichting & Pigliucci, 1998, modified)

Although environmental cues trigger phenotypic differences, the ability to respond is genetically based. Recent evidence suggests that part of this capacity is also due to epigenetic variations (Douhovnikoff & Dodd, 2015) or to somatic mutations that have been shown to segregate among ramets (Yu et al., 2020; see Box 1; Figure 1). If the latter is true, plasticity may interact with “hard‐wired” genetic changes that thus far have been neglected.

BOX 1 **Linking genetics to epigenetics**

The term *epigenetics* refers to all DNA and chromatin changes that can be inherited by the next generations and that do not involve changes in the DNA sequence (Bossdorf et al., 2008). These intrinsic mechanisms include methylation of cytosine residues, chromatin structure changes through chemical modifications of histone proteins, and a possible “crosstalk” between modifications at different levels (Holliday, 2006; Kouzarides, 2007). Overall, these modifications can be environmentally induced, promoting phenotypic plasticity through gene regulation and its heritability (*epigenetic plasticity*) (Feil & Fraga, 2012; Verhoeven et al., 2016). Since epigenetic marks can promote down‐ and up‐regulation of genes that can also be inherited to next generations, epigenetic alterations could be referred to as a regulatory machine, firstly for the acclimation response, and then through the fixation of that “epigenetic acclimation”, for a rapid adaptation (Dodd & Douhovnikoff, 2016; Richards et al., 2017). In this context, epigenetics could be the link between genetic diversity, phenotypic plasticity and the environment (Zhang et al., 2013). Another relevant issue in epigenetic mechanisms is related to the inheritance of histone modifications, as the possibility in plants to “remember” past stress events. This is already demonstrated for terrestrial clonal plants (Latzel et al., 2016; Verhoeven et al., 2016). Therefore, one of the most important epigenetic contributions in individual plasticity is the possibility to pass specific environmental information to the next generations and to regulate fast responses to ongoing environmental perturbations. This “learning process” could contribute to the accumulation of memory mechanisms, altering plant–environmental interactions in future generations. The epigenetic memory as plastic behavior seems to be partially responsible also for rapid phenotypic adjustments following fast environmental changes (Dodd & Douhovnikoff, 2016). For instance, *Arabidopsis thaliana* showed that a single genotype can display different epigenetic states, meaning that probably a widely epigenetic variation takes place between different ramets as a result not only of environmental conditions but also of the connection that exists among phenotypes, environment, and progenitors (Johannes et al., 2009). Recently, evidence about epigenetic mosaics within a genotype has been also shown in marine clonal plants, where epigenetics has been suggested as a key molecular mechanism enhancing phenotypic plasticity conferring thermal tolerance and the evolution of (pre‐) adaptive strategies (Marín‐Guirao et al., 2017, 2019). In particular, a case study performed on a clonal meadow of *Zostera marina* described epigenetics as the potential advantage to enhance beneficial phenotypic variations under environmental stressors without costs of clonal reproduction (Jueterbock et al., 2020). In seagrasses, new evidence pointed out the appearance of more tolerant phenotypes to contrast fast environmental shifts as a mechanism regulated by epigenetic rearrangement that occurs through genetic regulation (Jueterbock et al., 2020). Thus, the activation/inactivation of this regulatory machinery is strongly dependent on the environment triggering the existence of a stress memory with important implications for seagrasses exposed to future factors of stress (Nguyen et al., 2020). In *Posidonia oceanica*, differences in global DNA methylation has also been found among leaf tissue of different age in the same shoot, highlighting its role in the response to changes in environmental conditions (i.e., light availability and water temperature; Ruocco, De Luca et al., 2019; Ruocco, Marín‐Guirao et al., 2019). Additionally, an in silico gene–body–methylation approach showed that house‐keeping genes are hyper‐methylated, while genes with more inducible expression are widely hypo‐methylated (Entrambasaguas et al., *under review*).

Here, we focus on marine angiosperms (aka seagrasses) and describe the concept of phenotypic plasticity and its role in the face of rapid environmental changes as a potential way to overcome future environmental shifts. To do that, we focussed in particular on the most recent literature on seagrass responses to environmental stressors that has been critically analyzed also underlining the pros and cons of the technical approaches utilized. As an outlook, we suggest the most suitable approaches to analyze the role of plastic responses in seagrasses under global climate changes and local environmental stressors. A glossary of more specific terms utilized in this review is given in Table S1.

## 2 THE CONCEPT OF *PHENOTYPIC PLASTICITY*

The concept of “phenotypic plasticity” was applied for the first time by Nilsson‐Ehle (1914) in a case study of plant’ phenotypic changes resulting from different environments. Since then, the term itself evolved according to the development of new studies that document changes in environmental conditions, moving the scientific interest on organismal responses to environmental shifts. Not surprisingly, a broad literature defined concepts related to plasticity in plants during recent years. Different ways to define plasticity have been utilized, along with a variety of associated terms and more specific terminology (Kelly et al., 2012). Plasticity was also described with philosophical significance, as the ‘plastic nature’ of organisms or ‘plastic properties’ inherent to life. According to West‐Eberhard (1989), phenotypic plasticity can be defined as the ability of an organism to produce different phenotypes when it is exposed to different biotic and abiotic environmental conditions. In other words, a single genotype has the possibility to adjust its response to environmental changes, modifying its phenotypic state in terms of chemistry, physiology, morphology, and gene expression. The exposure to environmental variations enhances the development of different phenotypes (i.e. phenotypic plasticity) within and among individuals of the same population. The window of phenotype changes of a genotype along environmental variations defines its “phenotypic curve” or “reaction norm”, a basic and highly useful concept to understand the interrelations among phenotype, genome, and environment (Woltereck & Woltereck, 1909). Importantly, the reaction norm itself is under genetic control (Schlichting & Pigliucci, 1998) and can be defined as a function that relates the environment with the phenotype resulting from a particular genotype across an environmental gradient. This function can take any shape, and for continuously distributed traits, such as many physiological, morphological, and life‐history traits, it is typically visualized as a line or curve on a plot of the environment *vs* the phenotype (Gabriel & Lynch, 1992; Schlichting & Pigliucci, 1998). Evidently, deciphering more complex threshold/saturation type responses requires more than two measurements of the environment. Being a property of the reaction norm of single genotypes, plasticity is described by comparing the slope of the phenotype curve with the mean phenotypic value resulting from the external conditions. Consequently, the greater the slope of the curve, the more it deviates from the mean phenotypic value and the more the phenotype is plastic (Figure 1). Assessing the genetic basis of the reaction norm slope (i.e., phenotypic plasticity) is fundamental to explore the genotype–environment relation. In this regard, the genetic variation of a genotype is displayed by the “height” of the reaction norm plot. Thus, genotypes differing in terms of heights (genetic variation) and slopes (degree of plasticity) of their reaction norms are more likely to evolve (see next paragraph, Pigliucci, 2001; Schlichting & Pigliucci, 1998). Another important issue is that the shape of the reaction norm can be the result of a different organismal response along the biological hierarchy. Responses at the gene expression level, for example, may be plastic, that is, exhibit a strong slope when stress genes (HSPs) are activated (e.g., in seagrasses: Bergmann et al., 2010; Traboni et al., 2018). At the higher organizational level, however, this results in the maintenance or resilience of organismal function, for example photosynthesis, so essentially in a flat reaction norm with increasing stress (as in a generalist response) (Reusch, 2014).

In general, the analysis of processes involved in phenotypic plasticity and the possibility that such plastic responses might or might not be adaptive is complex. Currently, phenotypic plasticity is not unequivocally defined in seagrasses, and the approaches to assess the adaptive potential of phenotypic plasticity have not been standardized. Long‐life cycles and slow growth, which characterize most of the seagrass species, impede manipulative experiments and trans‐generation assessments. Additionally, advanced genetic tools, such as recombinant technologies (e.g., CRISP), are currently unavailable for all seagrass species and a complete sequenced genome is only available for two species, that is, *Zostera marina* (Olsen et al., 2016) and *Z. muelleri* (Lee et al., 2016).

## 3 THE GENETIC COMPONENT OF PHENOTYPIC PLASTICITY

Seagrass populations can be more resilient or resistant to environmental changes as the expression of individual or population plasticity. In general, the process can be addressed at two different but interconnected levels: genetic diversity displayed among genets, and number and distribution of genets at a particular location that can be summarized as genotypic diversity. Genetic diversity depends on the allelic variation and heterozygosity resulting from the sexual reproduction and the immigration of new genetic variants from other populations, whereas the genotypic diversity depends on the size structure and persistence of clones (or genets, consisting of many ramets) at a location, through vegetative propagation (i.e., clonal diversity) (Procaccini et al., 2007). Experimental studies have demonstrated that genetic and genotypic diversity of populations are a good proxy of population resilience and plasticity to changes (Ehlers et al., 2008; Hughes et al., 2008; Jahnke et al., 2015) since population reaction norm results in a broad sense from the amplitude of the reaction norms of single genotypes.

**FIGURE 2:**
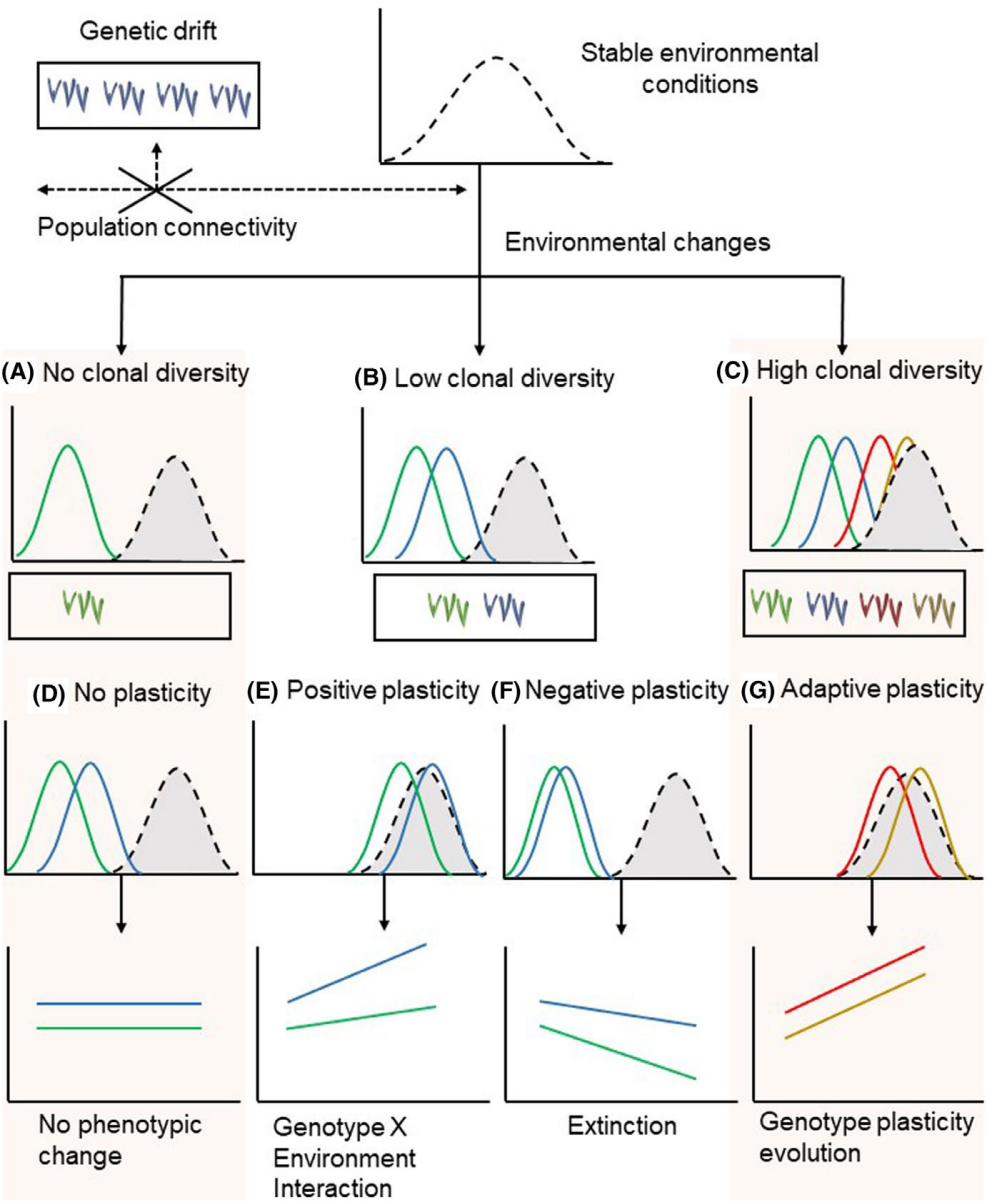
The role of genetic diversity and its effect on phenotypic plasticity in face of prompt environmental changes (see the text for more details)

Since seagrass genotypes can persist for a long time (>>100 years) as in long‐living species such as *P. oceanica* (Arnaud‐Haond et al., 2012) and *Z. marina* (Olsen et al., 2016), the genetic diversity among the genet level is maintained by the interplay between sexual reproduction and clonal growth (Arnaud‐Haond et al., 2020; Kendrick et al., 2012). This results in the formation of submerged meadows ranging from almost monoclonal to highly genetic diverse (e.g., *P. oceanica*: Arnaud‐Haond et al., 2012; *Z. marina*; Ferber et al., 2008; Figure 2).

Despite the importance of population size, low‐genetic variation has been found as a winner strategy in different plant species (e.g., clonal invasive species; Lambertini et al., 2010; Li et al., 2006), particularly in long‐lived ones (e.g., *P*. *oceanica*; Arnaud‐Haond et al., 2012; Ruggiero et al., 2002; *Z. marina*: Reusch et al., 1999). Population size (the level of genetic variation within populations) is considered a major constrain for the adaptation of natural populations to environmental changes, as the higher is the number of genotypes, the higher is the possibility that some of them can be positively selected (Bell & Gonzalez, 2009; Matesanz & Valladares, 2014). Effective population size can be decreased by the selection of plastic genotypes (adaptive phenotypes), in presence of rapid environmental changes, through changes of allele frequencies of specific loci and globally on the genome (Grenier et al., 2016).

Any adaptation to a new environment at population level is a process resulting from the natural selection of better‐suited genotypes across generations, changing the genetic composition of populations. This process can be slow, inducing an initial decline in population fitness and size and experiencing a subsequent increase once adaptive genotypes exhibit appropriate phenotypes (Hamilton & Miller, 2016; Valladares et al., 2014). The selection of these genotypes leads to changes in the frequency of alleles that confer greater fitness under the new altered conditions, promoting adaptive evolution (Grether, 2005). For this reason, the slope or shape of reaction norms is continuously evolving in most cases, rendering the mutually exclusive distinction of plasticity *vs*. adaptation meaningless (Schlichting & Pigliucci, 1998). The occurrence of somatic DNA mutations in single individuals can provide a readily available extra source of variation that was previously not considered and that can be maintained via clonal growth in long‐living genotypes (Whitham & Slobodchikoff, 1981). The role of somatic mutations in seagrasses was initially assessed by Reusch and Boström (2011) (Reusch & Boström, 2011). Recently, high somatic genetic variation was detected among ramets of a single genet of *Z. marina* plants (Yu et al., 2020).

Similar to genotypes within a population, populations from contrasting environmental conditions also showed different plasticity which is indicative of local adaptation (Sánchez‐Gómez et al., 2011). Thus, the existence of populations locally adapted to natural environments showing more adaptive genotype curves (i.e., reaction norms) results in population divergence in plasticity patterns representing an evolutionary potential for the species. In this sense, plasticity has the potential to drive population divergence as the environment changes (Pfennig et al., 2010). The capability of an individual to adapt and the timing of evolutionary adaptation is intrinsically related to its plasticity.

Even in species with high clonal persistence, such as *P. oceanica*, stochastic events of sexual reproduction and migration of genetic variants through populations via sexual propagules seem to suffice to promote genetic rearrangements and enhance selectively advantageous genetic variations (Arnaud‐Haond et al., 2014; Jahnke et al., 2015; Kendrick et al., 2012; Procaccini et al., 2007). The connectivity among populations depends on the existence of geographic or oceanographic barriers and the different features of dispersal vectors, that is, sexual or clonal propagules (e.g., Jahnke et al., 2016; McMahon et al., 2014; Serra et al., 2010). High‐resolution genetic data for the seagrass *Thalassia testudinum* along the western tropical Atlantic coasts revealed high‐genetic diversity as the result of high connectivity between subpopulations (i.e., gene flow) which in turn favored the appearance of different phenotypes (Bricker et al., 2011). Isolated meadows, instead, can progress toward genetic drift lowering allelic diversity and making populations even more fragile against changes in environmental conditions (Figure 2a). When environmental conditions change only more diverse populations could harbor genotypes able to face the new extreme conditions, while monoclonal or less diverse populations could disappear (Figure 2b–d). This is the reason for the higher sensitivity to environmental changes of marginal populations concerning central populations of the species distribution (e.g., Billingham et al., 2003). The alternative would be to move toward conditions that are more favorable or to adapt, requiring times that are not achieved against fast environmental changes as we are facing nowadays.

### 3.1 Genotype by environment interactions

Being a characteristic of individual genotypes, the amount of phenotypic variation across the environment describes the degree of genotype plasticity (genotypes by environment interactions – GxE; Li et al., 2018). Different reaction norms arise according to the degree of the interaction between individual genotypes and the environment (which is represented by the slope of each reaction norms in Figure 2). When environmental conditions change, populations with low genotypic diversity (Figure 2a,b) can react in different ways: (i) genotypes are stable and show no plastic behaviors (the reaction norms are parallel with the same shape, Figure 2d). Phenotypic changes do not occur, meaning that the mean of the phenotypic value of genotypes is enough to support environmental changes; (ii) genotypes re‐shape their phenotypes to the new environmental condition exhibiting positive phenotypic plasticity (Figure 2e). This results in different positive plastic responses depending on the individual genotype interaction with the new environmental factor; (iii) genotypes interact with the new environment showing phenotypic changes that are maladaptive or not able to accommodate new conditions (Figure 2f). Negative phenotypic plasticity could result in population extinction. Contrary, more diverse populations have the potential to exhibit more plasticity if most plastic genotypes bring phenotypes closest to the new optimum conditions (Figure 2e). Then, if the plastic response is positively correlated with plant fitness, phenotypic plasticity can evolve by natural selection (Valladares et al., 2014) leading to genotype plasticity evolution (Figure 2g).

## 4 PLASTIC RESPONSES TO RAPID ENVIRONMENTAL CHANGES: ACCLIMATION AND MIGRATION

**FIGURE 3:**
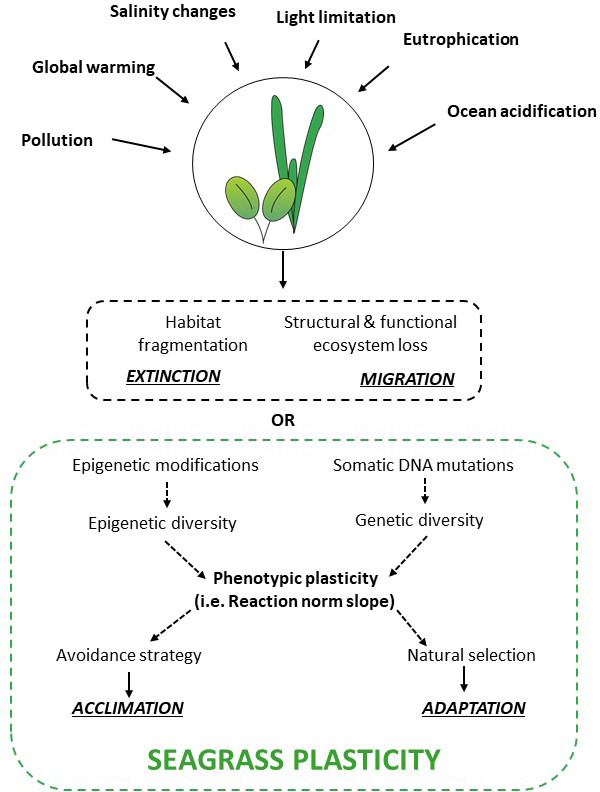
Representation of seagrass reactions to environmental changes. In the presence of environmental perturbations as global changes, seagrass survival is compromised, through habitat fragmentation and structural and functional ecosystem loss with consequent species extinction. Alternatively, intrinsic forces can increase their dispersal capacity to find more suitable environments (i.e., migration) or facilitate their persistence in the new environment through phenotypic plasticity. This adjustment to external conditions can be enhanced by epigenetic modifications or somatic DNA mutations, which increase epigenetic and genetic diversity, respectively. The resulting phenotype will favor the acclimation to the new environment and can be naturally selected. Thus, acclimation and adaptation are interrelated strategies of the seagrass plasticity representing intrinsic forces for their survival to future environmental changes

Currently, global environmental changes may be too fast to allow for selection and evolutionary changes to occur in long living species, resulting in mean decline in population fitness. Thus, the persistence of species in the age of global climate changes will mainly depend on their intrinsic abilities that facilitate their persistence under environmental shifts adjusting to new conditions (i.e., *acclimation capacity*) or increase their dispersal capacity to find a more suitable environment to which they are adapted (i.e., *movement capacity*, Figure 3).

Here, we refer to *acclimation capacity* as the most relevant short‐term response derived from pre‐existing phenotypic plasticity, which allows organisms to adjust to rapidly changing environments extending their tolerance ranges (De Los Santos et al., 2009; Sharon et al., 2009). Phenotypic responses to environmental changes occur at different organizational levels that may include highly specific developmental, morphological, and physiological adjustments enhancing survival and persistence in the novel environment (Bercovich et al., 2019; Zhang et al., 2014). The degree of plasticity, as stated above, is related to the slopes of reaction norms that are variable among individuals, populations, and species. Thus, the steeper is the slope of the reaction norm, the more an organism is able to acclimate to different environmental conditions, and the more it is plastic. The process of acclimation resulting from phenotypic adjustments to environmental cues can occur during the early development of an organism that persists also on the adult stage, or as reversibly acclimation occurring during the lifetime (Beaman et al., 2016). The continuous alignment of phenotypes to the environment involves associated costs to perform strategies for sensing and responding without affecting individual performances (Vialet‐Chabrand et al., 2017; Zimmerman, 2017). In fact, phenotypic plasticity is constrained by the energetic costs required for the sensory and regulatory mechanisms that ensure the processing of information and the development of the best phenotype–environment match (Gibbin et al., 2017). This is especially true for fast environmental changes and highly variable environments that force continuous prompt adjustment. The cost of switching the phenotype related to individuals and populations could be determinant for the ability of organisms to withstand environmental changes and to sustain the provision of ecological functions (Auld et al., 2010; Forsman, 2015; Murren et al., 2015). The high energetic costs involved in producing the optimum phenotype and the presence of a trade‐off diverting energetic costs to support other traits and/or functions (DeWitt et al., 1998) can also result in the appearance of phenotypes that are less fit to the new environment (non‐adaptive or maladaptive phenotypes) and hence not positively selected by evolutionary processes (see Figure 2 and previous paragraph; Palacio‐López et al., 2015). Non‐adaptive phenotypes would induce the decline of many species, including seagrasses, as fast global changes are currently increasing environmental stochasticity. However, costs related to phenotypic plasticity are currently under‐studied in seagrasses.

Short‐term responses occur through acclimatory mechanisms. In seagrasses, it has been described that these involve the modulation of gene expression profiles, which in turn depend on stress intensity, time of exposure to stressful conditions (Pernice et al., 2016; Ruocco et al., 2018), and morphometric plasticity in relation to geographical distributions and nutrient *status* (De los Santos et al., 2016; Soissons et al., 2018). At the individual level, plasticity can buffer environmental changes throughout the plant's lifetime, further increasing its tolerance to stress (e.g., short‐term acclimation to light conditions; Olesen et al., 2002). It has been shown that plants of the Mediterranean species *Posidonia oceanica* have plastic responses to different light conditions as a consequence of regulatory mechanisms that allow them to acclimate to low‐light environments (Dattolo et al., 2014; Mazzuca et al., 2009; Procaccini et al., 2017).

Plasticity at lower levels of the biological organization needs to be integrated at the level of individual and/or population fitness to evaluate how this influences the fitness and therefore alters the structure and the functioning of seagrass ecosystems. As an example, the stenohaline *P. oceanica* is able to thrive in environments with highly fluctuant salinity regimes thanks to high plasticity at the physiological (e.g., photosynthesis, carbohydrates metabolism) and morphological (e.g., plant size) levels (Marín‐Guirao et al., 2017). These adjustments permit the species to keep unaltered plant density and population growth rates, as a plastic response to maintain population fitness. Nevertheless, the reduced size of plants weakens the physical structure of the leaf canopy and thus, its functionality, affecting the provision of ecological services.

At the population—and ultimately species—level, plasticity can allow colonization and establishment in diverse habitats and therefore influences the species’ ecological breadth (Gimeno et al., 2009; Pigliucci, 2001; Sultan, 2003). In the presence of environmental stressors, plasticity could increase the dispersal capacity favoring the *migration* to more comfortable conditions or increase the reaction norm slope of a particular trait to cope with new environmental conditions. In this sense, organisms could adopt an escape mechanism to avoid unfavorable conditions due to environmental changes. Thus, the migration capacity can be described as an alternative strategy to local acclimation, which allows organisms to track more favorable conditions (Bulleri et al., 2018). It is important to emphasize that the movement capacity can be a consequence of trait plasticity when its reaction norm is defined by the interaction with the environment.

The migration capacity of seagrasses is related to clonal growth, sexual reproduction and dispersal of sexual propagules, and vegetative fragments (McMahon et al., 2014). This means that the motion capacity is very different among species and even within the same seagrass species given that the frequency of sexual reproduction, the dispersion of seeds (floating vs buried seeds), the rates of clonal elongation, and the persistence of plant fragments greatly vary among populations, let alone species (Orth et al., 2007). For instance, settling velocities of fragments are important for successful seagrass movements, which allow plants to disperse spatially. Thus, rapid settling capacities can be the result of an adaptive process that reduces the risks for plants of being away from theirs optimal habitats (Weatherall et al., 2016).

Overall, large and long‐lived species mainly rely on slow vegetative growth and have infrequent sexual reproduction events, which may potentially result in a reduced migratory success since they spread extremely slowly over large distances and seldom produce sexual propagules (McMahon et al., 2014). However, some species have shown high plasticity in reproductive phenology in response to environmental changes that increase their movement capacity. For instance, in terrestrial plants, it has been observed that different natural populations grown in common environments showed different flowering time in response to wet and dry conditions (e.g., *Brassica rapa*; Franks, 2011), a way to produce dispersal vectors (i.e., sexual propagules) and escape from the existing environment. *B. rapa* genotypes growing from seeds that experienced drought anticipate flowering in further dry conditions, in respect to seeds collected before the stressing event (Franks et al., 2007). This evidence suggests that the escape strategy adopted by these plants could be an indication of a rapid evolutionary shift to early flowering rather than the modification of the phenotypic state through trait adjustments (i.e., phenotypic plasticity). Thus, the potential to adopt plastic strategies is mostly the result of a trade‐off between avoidance (through phenotypic plasticity) and escape (through early flowering). Similar evidence was recently described for seagrasses, where flowering phenotypes resulted in response to warming (i.e., *Z. marina*, Blok et al., 2018; *P. oceanica*, Ruiz et al., 2018). Collecting and storing seeds from seagrass populations growing in different conditions would allow testing evolutionary processes in face of future environmental scenarios.

Small and more ruderal species, such as *Halophila stipulacea*, are able to migrate fast into new environments adjusting their dispersal ability through phenotypic plasticity. This species, a native from the Red Sea, rapidly spread and colonized new environments, as the Mediterranean and Caribbean seas, locally adapting through phenotypic changes, such as changing sex ratio (Nguyen et al., 2018; Winters et al., 2020). The rapid establishment and spread of this species in cooler regions are mediated by its great plasticity for shifting the thermal tolerance during the Mediterranean invasion (Georgiou et al., 2016; Nguyen et al., 2020; Wesselmann et al., 2020). Despite the migration being a valuable strategy to avoid species extinction, losers can be the native species that are potentially outcompeted by colonizing species (e.g., *H. stipulacea*; Winters et al., 2020).

It is noteworthy to mention that plasticity is an underlying attribute to these processes, which in turn are not mutually exclusive since acclimation, adaptation, and distributional changes are interrelated to some extend (Donelson et al., 2019; Kelly, 2019).

## 5 ASSESSING PHENOTYPIC PLASTICITY IN SEAGRASSES

The analysis of plasticity and the discrimination between adaptive or acclimation processes in plants has been mostly approached in model species, where the appropriate molecular and manipulative tools have been developed (Bossdorf et al., 2010; Matesanz et al., 2020). Seagrasses are a polyphyletic and unique group of plants, with convergent morphology due to constraining imposed by the adaptation to a fully submerged life in the marine environment (Les et al., 1997; Olsen et al., 2016). Sexual reproduction is adapted to the marine environment and its experimental manipulation has not been developed for most of the species. Hence, the dissection of the different drivers of plasticity can mostly be assessed based on indirect evidence. The complex and multidisciplinary information needed for disentangling plasticity components can be obtained through field observations, experimental manipulations, and laboratory approaches that are described in detail in the following paragraphs. The most recent seagrass literature has been reviewed to present the strength and weaknesses of each approach (Table 1; Table S2).

**TABLE 1:** Summary of pros and cons of approaches used to assess phenotypic plasticity in seagrasses (see the main text for more detail)

Approaches	Pros	Cons
Field observations	Inform about factors that potentially promote the evolution of phenotypic variation and how plasticity can contribute to evolutionary differentiation within species	Limited to observations
Field experiments	Quantify the degree of plastic responses, analyzing phenotypic changes in relation to the environment	Natural environmental variation leads to misleading interpretations
Mesocosm experiments	Simulate the effect of the stress factor of interest for analyzing intraspecific and interspecific responses and the genetic basis of phenotypic plasticity	Require sophisticated systems. Results cannot be automatically transferred to natural conditions
Reciprocal transplant experiments	Identify the genetic component of plastic responses	Sensitive to environmental forces and regional stressors
Common garden experiments	Allow discriminating the contribution of genetic and plastic effects comparing genetically distinct families or populations	Require long acclimation phases and an accurate experimental design

### 5.1 Field observations

Phenotypic plasticity, and in particular whether it is adaptive and which are the energetic costs involved, can be first approached by comparing performances between populations subjected to different environmental conditions (Forsman, 2015). Variation in single or multivariate trait plasticity along environmental gradients can inform about factors and conditions potentially promoting the evolution of phenotypic variation and give insights into how plasticity can contribute to evolutionary differentiation within species (Donelson et al., 2019).

Analysis of functional traits selected for plants combined with genetic data is a helpful approach to investigate genotype–environment interactions (Haseneyer et al., 2009). For instance, Maxwell et al. (2014) observed that physiological and morphological characteristics of *Zostera muelleri* varied along a gradient of water quality according to well‐known light acclimation responses. They also observed a consistent response in all meadows to a severe flooding event increasing freshwater run‐off along the gradient. Plants maintained population productivity unaltered (i.e., biomass, shoot, or leaf density alterations) through physiological adjustments, suggesting high phenotypic plasticity and a reaction norm with a large positive slope. In another example, the congeneric seagrass species *Z. noltii* showed the capacity to acclimate to local environmental conditions exhibiting different phenotypes in terms of mechanical and morphological traits during one growing season and across the latitudinal range of the species. The presence of stronger and stiffer leaves under oligotrophic as compared to more eutrophic conditions suggested that the species suffers in nutrient‐enriched environments without evolving a potentially adaptive phenotype (Soissons et al., 2017).

Phylogeny‐based comparative analyses can be used to infer the role of plasticity for evolutionary diversification among species and for speciation (Coyer et al., 2013; Olsen et al., 2004). Candidate genes that are indirectly related to environmental gradients, providing evidence of local adaptation, can be identified through genome‐wide transcriptomic analysis performed on wild populations (Jahnke et al., 2019), though the identification of real causation among genes and the environment is not trivial. This could be approached by combining genome‐wide analysis with manipulative stress experiments (e.g., Anderson et al., 2014).

The analysis of spatial variation across environments by comparing ecosystems and populations along gradients is a useful approach to extrapolate temporal dynamics and to infer about future ecosystem responses (i.e., space for time substitution; Fukami & Wardle, 2005). This is a valid approach, which states that environmental factors vary over time in the same way as they vary in space providing new opportunities to explore the potential success of plastic species (Buyantuyev et al., 2012). The analysis of samples along a wide spatial range allows to assess relationships between phenotypic variations and the environmental gradient without the constraints of time (Banet & Trexler, 2013). As showed by Bricker et al. (2011), *T. testudinum* individuals from different populations across north–south physiochemical environmental gradients in the Florida Bay was an effective method to discriminate plasticity as the main driver for phenotypic variations across sites. The space‐for‐time substitution approach is helpful not only to analyze populations’ plasticity through natural gradients and thus to assess long‐term consequences of human impacts, but also to infer temporal dynamics by comparing multiple sites with different disturbance gradients (Fukami & Wardle, 2005). For instance, Yang et al. (2018) showed, under different stress regimes, different degree of plasticity for physiological and morphological traits in *Z. marina* plants collected across regions that displayed diverse eutrophic gradients. New potential bio‐monitoring metrics, which may help the management of seagrass meadows in monitoring and predicting phenotypic variations, can derive from this kind of study.

### 5.2 Experimental manipulation of selected parameters

Observational studies can offer important insights in order to generate further hypotheses and testable predictions. However, demonstrating causal relationships and mechanisms, linking either variation in the capacity for plasticity itself or plasticity induced phenotypic variation to aspects of the individual or population fitness, is complex, as it requires experimental manipulation, replication, and controlled comparisons (Forsman, 2015).

The experimental manipulation of one or more environmental factors can be performed directly in the field or in the laboratory under controlled conditions. The last option requires a deep analysis of the relevant environmental factor to establish the correct experimental design, which in turn reflects the environmental variation that occurs under natural conditions. This is not an easy task, since many environmental factors act and interact with each other in natural conditions.

#### 5.2.1 Field experiments

Field experiments allow quantifying the degree of plastic responses, analyzing phenotypic changes in relation to the environment (Merilä & Hendry, 2014), and predicting shifts in species compositions under environmental changes (La Nafie et al., 2013). This can be realized through the artificial modulation of one or more factors, to compare control and treatment under natural environmental conditions, in order to investigate the potential drivers for the observed phenotypic changes. One of the major strengths of this approach is the inclusion of natural variability and processes that are difficult to reproduce under controlled conditions. In this respect, individual responses measured in situ provide more reliable results than those performed in the laboratory. Different studies have been carried out in the field, exploring phenotypic responses of seagrass species to single (e.g., Bité et al., 2007; Collier et al., 2012; Cox et al., 2015; Darnell & Dunton, 2017; Silva et al., 2013; Table S2) or to multiple environmental factors (e.g., Ceccherelli et al., 2018; La Nafie et al., 2013; Ravaglioli et al., 2017). For instance, Ruocco et al. (2018) showed that in *P. oceanica* plants, herbivory increases under nutrients addition, with a clear effect on seagrass productivity. In such environmental conditions, the species can enhance growth to compensate for the increase of herbivory, or can increase the accumulation of deterrent substances and the translocation of nutrients to underground tissues to protect them against external pressures (Alcoverro & Mariani, 2005; Ruocco et al., 2018; Sánchez‐Sánchez & Morquecho‐Contreras, 2017).

Tuya et al. (2019) assessed the tolerance of *C. nodosa* to low‐light levels across different populations located in the Canary Islands and the Mediterranean Sea by manipulating the light intensity directly in the field. Results demonstrated biogeographical variability among populations in the degree of shading tolerance, with Canary Island populations being less tolerant in respect to the others. As suggested by authors, the lower plasticity of Canary Island populations can be related to the lower genetic diversity of these populations, living at the range edges of species’ distribution. Salo et al. (2015) also found different gene expression and physiological performance of *Z. marina* genotypes to light reduction. The experimental manipulation in the field offers also the opportunity to study plastic responses of plants locally adapted to particular environmental conditions. In order to model the response to eutrophication in a future ocean acidification scenario, Ravaglioli et al. (2017) evaluated the performances of *P. oceanica* plants adapted to long‐term acidification by exposing them to in situ nutrient enrichment. The field experiment revealed that the increased CO_2_ benefits plants facilitating the absorption and assimilation of nutrients.

Although experimentation in the field is helpful for quantifying the plasticity in the response to environmental stressors, the natural environmental variability can lead to misleading interpretations of the specific drivers responsible for the resulted phenotypic changes. Additionally, these experiments provide results that are difficult to replicate and compare with similar studies because regional stressors and biotic interactions may modify the final outcome (e.g., Garrote‐Moreno et al., 2016).

#### 5.2.2 Mesocosm experiments

One of the main advantages of performing experimental manipulations under controlled conditions is the possibility to simulate the effect of the stress factor of interest, isolating it from all the other variables that are naturally occurring, and to analyze intraspecific and interspecific responses. Additionally, controlled experiments offer the opportunity to evaluate the degree of phenotypic plasticity in the form of a genetically determined reaction norm. An example from terrestrial plants refers to the manipulation of temperature, utilized for assessing thermal tolerance variability across latitudes (Molina‐Montenegro & Naya, 2012). In this case, the authors measured the phenotypic plasticity of an invasive species (*Taraxacum officinale*) to different environmental temperatures, confirming that higher thermal tolerance at higher latitudes is related to an improved phenotypic expression. Different studies performed under laboratory conditions assessed phenotypic plasticity of seagrass species, such as the mesocosm experiments performed on the most abundant Mediterranean species, *Posidonia oceanica* and *Cymodocea nodosa*. These studies have confirmed that these two species have different tolerance to hypersaline stress (i.e., *C. nodosa* > *P. oceanica*), consistent with their physiological and morphological plasticity (Piro et al., 2015; Sandoval‐Gil et al., 2012, 2014). Furthermore, *C. nodosa* also showed higher tolerance and higher plasticity to warming, possibly related to the tropical affinity of the genus (Marín‐Guirao et al., 2018; Olsen et al., 2012; Tutar et al., 2017). Controlled experiments also allow the manipulation of multiple stressors simulating realistic environmental changes affecting coastal marine habitats (Artika et al., 2020; Egea et al., 2018; Pazzaglia et al., 2020; Viana et al., 2020). Through the manipulation of temperature and nutrients concentration, Ontoria et al. (2019) investigated individual and population responses in *C. nodosa* plants. Different phenotypes arose depending on the interaction among temperature‐ammonium and temperature‐organic carbon suggesting that the exposure to multiple stressors triggers phenotypic responses in relation to stress‐specific thresholds. The analysis of the recovery after stressing conditions, allowed to point out contrasting resilience abilities of seagrass populations living in different environments, as a result of their adaptation to local climatic conditions (e.g., Franssen et al., 2011; Winters et al., 2011; Table S2). This represents an important advantage of experimental manipulations, as offers the possibility to understand if plants are able to turn back to their original natural state after extreme events providing new insights into the long‐term survival of seagrasses to environmental changes.

### 5.3 Transplantation experiments

Transplantation experiments fall into two distinct approaches. A reciprocal transplant experiment entails the movement of phenotypes between contrasting natural environments along with on‐site transplantation controls. In common garden experiments, genotypes coming from different environments are planted into the common environmental conditions of a single site.

#### 5.3.1 Reciprocal transplant experiments

This experimental approach allows for a direct test of local adaptation by comparing two sites with each other (Kawecki & Ebert, 2004). Thus, provided proper acclimation and control for carry‐over effects (see below), a potential genetic component of the plastic response (as reaction norm) can be quantified by comparing the phenotypic performances of transplants in native vs. foreign environments. Local adaptation and plastic abilities of different populations can be addressed using two different comparisons. First, local populations can be compared within habitats, that is, “local” vs “immigrant” design; second, plants can be compared across habitats, that is, “home” vs “away” design (Svensson et al., 2019). The final expectation of such experimental conditions is that plants perform better in their “home” environment in respect to the “away” ones, showing direct indications of a local adaptation. In this case, the degree of plasticity of genotypes locally adapted to their home site and transplanted to reciprocal environments within their environmental tolerance range can be assessed. A recent review summarizing 75 years of plant experiments on local adaptation revealed that indeed, local populations almost always showed higher performance than non‐local ones, especially in traits related to reproductive output, suggesting a notably local adaptation in terrestrial plants (Baughman et al., 2019).

Factors other than local adaptation can affect transplants performance. Evans et al. (2018) designed a reciprocal transplantation experiment of two genetically and geographically distinct populations of *P. australis* in southeastern Australia. They assessed local adaptation by comparing plant productivity of low‐ and high‐genetic diversity meadows using the “home” vs “away” approach. After 6 months, they found higher survival rates and productivity for high‐genetic diversity plots, which outperformed less genetically diverse plants both at home and away sites. This means that more genetically diverse plots included also more plastic genotypes that performed better than less diverse plots, allowing them to survive after transplantation. This high genetic demonstrated that both high‐genetic diversity and local adaptation play a crucial role in enhancing transplant success (Hämmerli & Reusch, 2002; Jahnke, Serra, et al., 2015; Procaccini & Piazzi, 2001; Reusch et al., 2005; Reynolds et al., 2012; Williams & Davis, 1996; Williams, 2001).

A reciprocal transplantation approach has also been used to evaluate seagrass short‐term acclimation along environmental gradients. Sharon et al. (2009) transplanted shoots of *H. stipulacea* between the depth extremes of its distribution, to evaluate the plastic response to different irradiance regimes (Table S2). After 2 weeks of exposure to reciprocal environments, they found fast changes in photosynthetic performance supporting the high plasticity of the species.

The long‐term maintenance of field experiments can be jeopardized by environmental forces (storms, salinity, and temperature fluctuations), regional stressors (anchoring boats and anthropic inputs), or other technical problems (van Katwijk et al., 2009). As an alternative, reciprocal transplant experiments in controlled conditions are a valid tool to overcome logistical issues with transplantation in the field that also includes the risk of introducing as yet unknown pathogens over longer distances. In *P. oceanica*, plants from shallow and deep environments were transplanted in individual pots and exposed to their reciprocal light regimes in a controlled mesocosm approach (Dattolo et al., 2017). *P. oceanica* genotypes showed some degree of photo‐physiological and morphological plasticity. Nevertheless, after several weeks under reciprocal light environments, genotypes showed performances that were similar to those shown by plants from their original depth, suggesting local adaptation to their home environment.

#### 5.3.2 Common garden experiments

Common garden experiments are particularly relevant to investigate the nature of plastic responses and to discriminate the contribution of genetic and plastic effects on phenotypic variation. In fact, these experiments allow comparing distinct genotypes or populations from different environments by growing them under identical environmental conditions (De Villemereuil et al., 2016; Merilä & Hendry, 2014). This approach is commonly used to test for local adaptation, as it enables to unravel the genetic basis of phenotypes from different populations excluding the effects of the corresponding environments (Cruz et al., 2019; De Villemereuil et al., 2016; Lepais & Bacles, 2014; Vermaat et al., 2000).

In seagrasses, Franssen et al. (2011) performed a common garden stress experiment to assess transcriptomic profiles of *Z. marina* populations from two contrasting thermal environments (Venice Bay, Italy, vs. Limfjord, Denmark) to a simulated heat wave. They found a strong divergence in terms of gene expression profiles between populations only in the recovery phase, while the immediate stress response was similar and showed the typical heat shock protein‐encoding genes with overexpression. This was consistent with local adaptation to the local natural thermal environment. One caveat of such studies is that even under a long acclimation phase of about 1 month, this may not be sufficient to overcome long‐term acclimatization to the home environment. We can thus not fully conclude that observed stress responses resulted from a genetically based adaptation (as stated by Bergmann et al., 2010; Winters et al., 2011; Table S2). One way forward to overcome such limitations is raising the experimental plants from seeds. This was done in seagrasses, for the first time to our knowledge, in eight populations across the distribution range of the seagrass *C. nodosa*. Seeds were germinated and subsequently grown for sixteen months in a common garden before being exposed to two marine heat waves of different intensity (Pereda‐Briones et al., *under review*). The positive relationship observed between the resilience and local thermal regimes of the studied populations strongly evidenced local adaptation of the populations to their thermal regime. Such studies provide strong evidence for the existence of underlying genetic variation resulting from divergent selection, representing the evolutionary potential of the species within the frame of global warming, although the attainable rates of change remain obscure (Reusch & Wood, 2007). This “adaptive transgenerational plasticity” is not only the result of the development of specific traits in response to environmental stresses passed from parental individuals to the progeny, but also the inheritance of regulatory epigenetic machinery enhancing offspring to activate regulatory mechanisms under the same stresses (King et al., 2018). Despite the relative long acclimation imposed on plants under common conditions, it remains still difficult to conclude on the genetic and/or epigenetic basis of the observed plasticity. Long acclimation phases and phenotypic responses of individuals under common conditions over one or more generations are necessary to test for adaptive traits in order to reset plants’ experiences of their place of origin (as in terrestrial model species; Raabová et al., 2007; Watson‐Lazowski et al., 2016). However, as stated above, the reproduction of seagrasses under controlled conditions is challenging, and life cycles are often too long to allow experimentation over multiple generations.

Common garden experiments can also be designed based on a space‐for‐time substitution approach. A case in point is the study by Winters et al. (2011) that compared plant responses to a heat wave originating from three populations of *Z. marina* across a latitudinal thermal gradient. The differential thermal response in terms of growth and photo‐physiology was consistent with local adaptation and could be integrated into seagrass models to predict the future persistence of this species in different regions affected by climate changes.

Some common garden experimental designs are a merger of all approaches described above (Jueterbock et al., 2016; Marín‐Guirao et al., 2018). Jueterbock and colleagues tested temperature adaptation of *Z. marina* populations collected from contrasting and phylogenetically independent thermal clines (North vs South in Mediterranean and Atlantic areas), using a common garden experiment combined with a space‐for‐time substitution design in anticipation of rapid ocean warming predicted for the next decades. Upon exposure of plants to a marine heat wave, full transcriptome profiles were obtained and mapped onto the genome. Results revealed a stronger adaptive transcriptomic differentiation between the Mediterranean and the Atlantic samples that is likely due to the reduced gene flows that characterized the smaller and isolated Mediterranean populations, favoring adaptive differentiation (Olsen et al., 2004; Procaccini et al., 2007).

## 6 FUTURE PERSPECTIVES: ENHANCING PLASTICITY FOR BOOSTING SEAGRASS ADAPTATION

Ascertained the importance of phenotypic plasticity and its role in driving short‐term responses and evolution, it is now necessary to explain how all this information can be integrated into seagrasses research. In the framework of conservation and restoration management, understanding the phenotypic plasticity of selected meadows to restore a disrupted habitat strongly boosts the success of restoration plans (Falk et al., 2001; Paulo et al., 2019). In fact, the selection of highly plastic and tolerant/resilient genotypes of foundation species could be a valid approach to restore marine ecosystems (Abelson et al., 2020; Coleman et al., 2020; Kettenring et al., 2014; van Katwijk et al., 2009). Selected genotypes should present a set of positive traits in order to increase their plasticity for successfully facing coming fast environmental changes.

In order to support the restoration, better performing genotypes can not only be identified and selected but can also be experimentally manipulated. A possible way is to use gene‐editing approaches, though their ethical implications are currently under debate (Rodriguez, 2016). After the identification of genes that directly affect seagrass ability to thrive in a changing climate, genetic engineering techniques, such as CRISPR (Clustered Regularly Interspaced Short Palindromic Repeats), can be used to produce genotypes with higher plasticity and the ability to acclimate and adapt to strong and stochastic environmental changes (Scheben et al., 2016).

Another way, which does not involve genetic manipulation, is the experimental hardening. The terms “hardening” or “priming” define phenomena that induce temporally limited environmental stimulus in order to prepare and modify the response to future stress (Hilker et al., 2016). This is a well‐known concept among botanists, which used to harden plants taking advantage of their ability to “remember” their ancestral environments via phenotypic plasticity, revealing a mechanism by which past experience affects future evolution (Gibbin et al., 2017; Ho et al., 2020). The capability of genotypes to save memory of past stress events and to better perform when the stress re‐occurs has recently been observed in seagrasses (i.e., *P. australis* and *Z. muelleri*, Nguyen et al., 2020). This process is one element of “assisted evolution” strategies to promote individual and population resilience against environmental changes without genetic manipulation constraints (Filbee‐Dexter & Smajdor, 2019). Genotypes with improved resistance (i.e., hardening response, Bruce et al., 2007) can represent preferential material for the restoration of endangered or disturbed populations. In terrestrial studies, the ability to “remember” past stressful events is currently investigated for model crop species, especially through the assessment of epigenetic modifications induced by the exposure to stress (Liu et al., 2015). Although the field of *ecological epigenetic*s is gaining momentum, due to the application of increasingly specific and sophisticated molecular techniques (Ay et al., 2014; Bossdorf et al., 2010; Popova et al., 2013; Rendina Gonzàlez et al., 2018; Richards et al., 2017), the study of the epigenetic “stress memory” is still at the beginning, for both marine and terrestrial plants.

## 7 CONCLUSIONS

Our main goal was to present an overview on the importance of plasticity in the face of rapid environmental changes for a group of marine plants with long generation times owing to clonality that lives in an environment with very steep environmental gradients, subject to alarming rates of global change. The rapid occurrence of global changes forces marine plants to react in order to prevent population declines. Species react acclimating to new conditions, through phenotypic plasticity, evolutionary adaptation, or migration (Bulleri et al., 2018). The acclimation abilities as one major form of phenotypic plasticity are widely explored in seagrasses’ studies (Bité et al., 2007; Dattolo et al., 2017; Duarte et al., 2018; Maxwell et al., 2014). This acclimation process can be based on genetic and epigenetic processes, the last fostering rapid adaptive evolution (Douhovnikoff & Dodd, 2015), but it is so far unstudied in seagrasses. Equally, unstudied is the adaptive significance of a large degree of standing somatic genetic variation detected in seagrass clones that could be the basis for adaptation within a genet or clone (Yu et al., 2020). The adaptation occurs through natural selection and requires too long times to react in the face of rapid changes. Nevertheless, the selection of more plastic genotypes could prevent population declines, as they are more likely to contrast dynamic changes (Bricker et al., 2011; Table S2).

We explored several main approaches that allow us to infer the nature of plastic responses to global changes and discussed pros and cons. The experimental approaches implemented in seagrass studies, whether performed with controlled or field experiments and space for time designs, were instrumental for exploring the basis of plasticity. One future avenue is clearly more multi‐factorial experiments that would be required to understand seagrass responses under more realistic present and future scenarios. Another important way forward is the integration of different phenotypic and genomic approaches to study the interaction among the genetic and plastic components of phenotypic variation, including the study of epigenetic mechanisms. Considering the importance that plasticity may have in response to rapid environmental changes, future promising research in seagrasses should involve the analysis of relationships between gene expression profiles resulting from environmental stresses and epigenetic regulatory machinery. The majority of seagrass studies employing molecular approaches involve gene expression and transcriptomic analysis, while being limited to few species and mostly related to thermal and light responses (Davey et al., 2016; Gu et al., 2012; Marín‐Guirao et al., 2017; Procaccini et al., 2017; Tutar et al., 2017). We also observed that many recent transcriptomic studies in response to environmental stressors lack consideration of molecular elements that may have strongly regulatory roles in stress responses, such as transposable elements and micro‐RNA (miRNAs) (e.g., Barghini et al., 2015). The improvement of molecular approaches in seagrasses could play a crucial role not only in studying their plasticity but also in digging on the basis of stress memory and on its potential evolutionary role under global climate changes (Chinnusamy & Zhu, 2009; Lämke & Bäurle, 2017).

In conclusion, we strongly suggest that the evaluation of plastic adaptive responses should be moved from a local to a global scale. The future implementation and evolution of seagrass observatories will foster this process. Next‐generation marine observatories should make it possible to collect multivariate time series synchronously in different sites or regions and to exploit the information by integrating data through multivariate statistics and/or machine‐learning algorithms (Crise et al., 2018; Danovaro et al., 2016). Real‐time multivariate monitoring in seagrass observatories will enable assessing environmental and seagrass trait changes and inferring adaptive potential of the observed processes in seagrass populations.

## CONFLICTS OF INTEREST

There is no conflicts of interest to declare.

## Supporting information

Table S1

Table S2

## Data Availability

Data sharing not applicable to this article as no datasets were generated or analyzed during the current study.
