# Supplementary material for: Phenotypic plasticity under rapid global changes: The intrinsic force for future seagrasses survival
Source: Evol Appl. 2021 Apr 4;14(5):1181–201. doi: 10.1111/eva.13212 (PMC8127715; doi:10.1111/eva.13212)
Supplement: Supplementary file 1 — Table S1 [file EVA-14-1181-s002.pdf]

Table S1. Glossary of main terminology and definitions

**Phenotype**	The expression of a trait as a result of both genetic and environmental changes.
**Genotype**	The genetic makeup (i.e. complete genome) of a single organism.
**Adaptive phenotype**	The phenotype resulting from long-evolutionary mechanisms that allows organisms to optimally respond to environmental changes
**Non-adaptive phenotype**	The phenotype resulting from long evolutionary mechanisms that is not optimal for the new environment
**Reaction norm**	The function which describe a curve that relates the environment to a phenotype resulting from the interaction between a particular genotype and the environment. Different shapes of this function describe the degree of plasticity of a single genotype across the environment.
**Acclimation**	The ability of a single organism to adjust its morphology and physiology to environmental changes. This process occurs in short time improving performances in a changing environment. The degree of acclimation is closely related to phenotypic plasticity or the range tolerance of an organism.
**Epigenetic changes**	The modifications occurring at DNA level without altering DNA sequence. Epigenetic marks includes DNA methylation (DNA base modification through the addition of a methyl group) and histone modifications that alter DNA accessibility and chromatin structure.
**Epigenetic plasticity**	Epigenetic modifications induced by environmental cues with the ability of reprogramming gene expression. In the case of environmental shifts, these modifications trigger the expression of new phenotypes that can or cannot be advantageous.
**Assisted evolution**	Active intervention to accelerate the rate of naturally occurring evolutionary processes, including the selection of highly plastic

	genotypes, hardening and genetic manipulation, in order to improve performances under rapid environmental changes.
**Stress memory**	The ability to "remember" past stressful events. The capacity of plants submitted to a stress exposure to store information and to better respond to a new stress.
