# Supplementary material for: Phenotypic plasticity under rapid global changes: The intrinsic force for future seagrasses survival
Source: Evol Appl. 2021 Apr 4;14(5):1181–201. doi: 10.1111/eva.13212 (PMC8127715; doi:10.1111/eva.13212)
Supplement: Supplementary file 2 — Table S2 [file EVA-14-1181-s001.docx]

**Table S2. List of studies addressing plasticity in seagrasses (examples from the text)**

List of studies addressing plasticity in seagrasses and discussed in the text. The table lists the experimental design that has been applied, analyzed traits, the environmental factor, form of plasticity addressed, main results and the corresponding reference. Experimental design: FO = Field observation; TE= Transplant experiment; ME = Mesocosm experiment; FE = Field experiment; CGE = Common garden experiment; SFT = Space for time; CGE/STS = Common garden experiment with a Space for time substitution design. Plasticity: L_Adp = Local adaptation; L_Acc = Local acclimation; Phev = Phenotypic variation;

| *Species* | *Life-style, traits* | *Exp.design* | *Traits* | *Environm. factor* | *Plasticity* | *Result* | *Reference* |
| --- | --- | --- | --- | --- | --- | --- | --- |
| *Posidonia oceanica* | Po: High fruit dispersal; High clone longevity; Low growth | TE | Genetic diversity; Morphology; Growth | - | Phev | Plants transplanted from a donor population with the highest genetic polymorphism showed the best performances | Procaccini & Piazzi, 2001 |
| *Zostera marina* | Zm: High fruit dispersal; High clone longevity; High growth | TE-ME | Genetic diversity; Growth | - | Phev | Genetic diversity contributes to eelgrass population viability | Williams 2001 |
| *Zostera marina* | Zm: High fruit dispersal; High clone longevity; High growth | TE | Genetic diversity; Biomass | - | L_Adp | Genets produced more biomass in their home site due to local adaptation | Hämmerli et al. 2002 |
| *Thalassia hemprichii; Thalassodendron ciliatum; Cymodocea rotundata; Cymodocea serrulata; Halodule uninervis; Syringodium isoetifolium* | Th, Tc: Medium fruit dispersal; Medium clone longevity; Medium growth  Cr; Cs; Hu: Low fruit dispersal; Medium-low clone longevity; High growth  Si: High fruit dispersal; Medium clone longevity; Medium growth | FE | Leaf elongation; Nitrogen content | Nutrients | Phev | Interspecific variability in terms of phenotypic plastic responses to defoliation and nutrients budget | Alcoverro et al. 2005 |
| *Zostera marina* | Zm: High fruit dispersal; High clone longevity; High growth | FE | Genotypic diversity; Biomass; Shoot density; Leaf shoots; Epifaunal abundance and diversity | - | Phev | Genotypic diversity increased the rate of recovery after perturbation playing a role similar to species diversity and functionality | Reusch et al. 2005 |
| *Halophila ovalis; Zostera capricorni* | Hs: High fruit dispersal; Low clone longevity; High growth  Zc: High fruit dispersal; Medium-low clone longevity; High growth | FE | Chlorophyll a fluorescence | Light | Phev/L_Adp | Interspecific variability in terms of photo-acclimation to shading | Bité et al. 2007 |
| *Halophila stipulacea* | Hs: High fruit dispersal; Low clone longevity; High growth | TE | Chlorophyll a fluorescence; Chlorophylls content | Light | L_Adp | Fast changes in photosynthetic activity supporting the high plasticity of the species | Sharon et al. 2009 |
| *Zostera marina* | Zm: High fruit dispersal; High clone longevity; High growth | ME | Gene expression | Temperature | Phev/L_Adp | The combined effects of warming and ammonium were much worse than those of each stressor in isolation | Bergmann et al. 2010 |
| *Thalassia testudinum* | Tt: Medium fruit dispersal; Medium clone longevity; Medium growth | SFT | Genetic diversity | - | L_Acc | Phenotypic plasticity, rather than genotypic adaptation is the main driver for morphological variations. | Bricker et al. 2011 |
| *Zostera marina* | Zm: High fruit dispersal; High clone longevity; High growth | CGE | Gene expression | Temperature | Phev/L_Adp | Local thermal adaptation to contrasting environments influences thermal responses | Franssen et al. 2011 |
| *Zostera marina* | Zm: High fruit dispersal; High clone longevity; High growth | CGE/STS | Chlorophyll a fluorescence; Gene expression; | Temperature | L_Adp | Differential thermal response related to local adaptation can be integrated in seagrass models to predict their future persistence | Winters et al. 2011 |
| *Zostera noltii* | Zn: Low fruit dispersal; Low clone longevity; High growth | FO | Morphology; Mechanical traits | Latitudinal gradient; Nutrients | Phev/L_Acc | Mechanical and morphometric traits changes across latitude and depend on local nutrient conditions | Collier et al. 2012 |
| *Posidonia oceanica Cymodocea nodosa* | Po: High fruit dispersal; High clone longevity; Low growth  Cn: Low fruit dispersal; Medium-low clone longevity; High growth | ME | Biomass and growth | Temperature | Phev | Interspecific responses to warming | Olsen et al. 2012 |
| *Cymodocea nodosa* | Cn: Low fruit dispersal; Medium-low clone longevity; High growth | ME | Respiration and photosynthetic rate; Pigment content; Leaf light absorption; Growth and survival | Salinity | Phev | Higher salinity concentrations reduced photosynthetic performances. | Sandoval-Gil et al. 2012 |
| *Zostera marina; Cymodocea nodosa* | Zm: High fruit dispersal; High clone longevity; High growth;  Cn: Low fruit dispersal; Medium-low clone longevity; High growth | FE | Chlorophyll a fluorescence; Chlorophylls content; Carbohydrates; Malondialdehyde and phenol contents; Soluble proteins | Light | Phev/L_Acc | Interspecific phenotypic responses to light | Silva et al. 2013 |
| *Posidonia oceanica; Cymodoca nodosa* | Po: High fruit dispersal; High clone longevity; Low growth  Cn: Low fruit dispersal; Medium-low clone longevity; High growth | ME | Morphology; Leaf-water relations variables; Carbohydrates; Chlorophylls content; Chlorophyll a fluorescence | Salinity | L_Adp | Inter- and intraspecific divergences derives from different degree of plasticity that lay a role to respond to environmental stressors | Sandoval et al. 2014 |
| *Zostera muelleri* | Zm: Low fruit dispersal; Medium-low clone longevity; High growth | FO | Carbohydrates; Isotopic ratio; Cholorophyll content; Fluorescence | Light and Nutrients | L_Acc | Different morphological and physiological characteristic along a water quality gradient | Marxwell et al. 2014 |
| *Posidonia oceanica* | Po: High fruit dispersal; High clone longevity; Low growth | ME | Respiration rate; Net calcification; Chlorophyll a fluorescence; Epiphytes composition | Acidification | Phev | Acidification increased plant productivity despite the partial loss of epiphytes | Cox et al. 2015 |
| *Zostera noltii* | Zn: Low fruit dispersal; Low clone longevity; High growth | TE | Genetic diversity | - | Phev/L_Adp | Low genotypic richness after transplantations due to low plasticity of donor plants | Jahnke et al. 2015a |
| *Zostera marina* | Zm: High fruit dispersal; High clone longevity; High growth | ME | Morphological Traits; Growth rate; Photosynthetic performance; Nutrient content; Non-Structural carbohydrates; | Nutrients and Salinity | Phev | Synergistic effect under high nutrient concentrations and low salinity levels, which was different under the exposure to single stressors | Villazan et al. 2015 |
| *Zostera marina* | Zm: High fruit dispersal; High clone longevity; High growth | FE | Morphology; Carbohydrates: Gene expression | Light | L_Adp | Plasticity is related to genetic diversity | Salo et al. 2015 |
| *Cymodocea nodosa* | Cn: Low fruit dispersal; Medium-low clone longevity; High growth | ME | Chlorophyll a fluorescence; Leaf-water relations variables; Proteomic expression | Salinity | L_Adp | Physiological tolerance to salinity stress evidence proteomic and physiological adaptation to the selected ionic and osmotic stressful conditions | Piro et al. 2015 |
| *Posidonia australis; Posidonia*  *Sinuosa; Amphibolis antarctica; Amphibolis griffithii* | Pa: High fruit dispersal; High clone longevity; Low growth  Aa; Ag: Low fruit dispersal; Medium clone longevity; Medium growth | ME | Total ion concentrations | Salinity | Phev/L_Acc | Leaf ion concentrations increased at higher salinity conditions with ion ratio differences among species and native environment of plants. | Garrote-Moreno et al. 2016 |
| *Zostera marina* | Zm: High fruit dispersal; High clone longevity; High growth | CGE/STS | Gene expression | Temperature | L_Adp | Adaptive transcriptomic differentiation that is likely due to reduced gene flows favoring adaptive differentiation | Jueterbock et al. 2016 |
| *Thalassia testudinum* | Tt: Medium fruit dispersal; Medium clone longevity; Medium growth | FE | Morphology; Leaf nutrients content; Flower production and | Nutrients | Phev | High nutrients concentration decreased sexual reproduction rather than somatic growth | Darnell et al. 2017 |
| *Zostera noltii* | Zn: Low fruit dispersal; Low clone longevity; High growth | FO | Morphology; Mechanical traits | Nutrients | L_Acc | Different leaf mechanical traits in relation to morphometric plasticity and the nutrient status. | Soissons et al. 2017 |
| *Posidonia oceanica* | Po: High fruit dispersal; High clone longevity; Low growth | FE | Gene expression; Chlorophylls content; Moprphology: Epiphytes assemblage | Acidification | L_Acc | Effects of acidification on plants depend on local nutrients concentration and on acclimation capacity | Ravaglioli et al. 2017 |
| *Posidonia oceanica Cymodocea nodosa* | Po: High fruit dispersal; High clone longevity; Low growth  Cn: Low fruit dispersal; Medium-low clone longevity; High growth | ME | Gene expression | Temperature | L_adp | Intraspecific thermal responses to heat stress. Shallow plants performed better than deep ecotypes. | Tutar et al. 2017 |
| *Posidonia oceanica* | Po: High fruit dispersal; High clone longevity; Low growth | TE | Genotypization; Gene expression; Chlorophyll a fluorescence; Chlorophylls content; Morphology; Carbohydrates; | Light | Phev/ L_Adp | Phenotypic plasticity allows plants to acclimate to reciprocal environmental conditions through plastic regulation supporting the role of local adaptation to different depths | Dattolo et al. 2017 |
| *Posidonia oceanica* | Po: High fruit dispersal; High clone longevity; Low growth | FE | Morphology; Growth; Physiological and biochemical variables; Gene expression | Nutrients and burial |  | Synergic effects of nutrients and sediment burial | Ceccherelli et al. 2018 |
| *Zostera marina* | Zm: High fruit dispersal; High clone longevity; High growth | SFT | Morphology; Total carbon and total nitrogen; Chlorophylls content; Meta-analysis | Nutrients | Phev | Analysis of phenotypic plasticity across different regions is a valid method to develop bio-monitoring metrics. | Yang et al. 2018 |
| *Posidonia australis* | Pa: High fruit dispersal; High clone longevity; Low growth | TE | Genotypization; Genetic diversity; Morphology; Nitrogen content and total phenols; Carbohydrates | - | L_Adp | High genetic diversity and local adaptation play a crucial role in enhancing transplant success | Evans et al. 2018 |
| *Cymodocea nodosa* | Cn: Low fruit dispersal; Medium-low clone longevity; High growth | ME | Morphological Traits; Growth rate; Photosynthetic performance; Nutrient content; Non-Structural carbohydrates; | Temperature; CO2; Nutrients | Phev | Combined responses were larger than the sum of individual responses with antagonistic and synergistic effects | Egea et al. 2018 |
| *Posidonia oceanica Cymodocea nodosa* | Po: High fruit dispersal; High clone longevity; Low growth  Cn: Low fruit dispersal; Medium-low clone longevity; High growth | ME | Photosynthetic performance; carbon balance; carbohydrates content; growth and mortality | Temperature | L_adp | Plant responses showed geographic heterogeneity. Warm plants were more tolerant than cold plants. | Marin-Guirao et al. 2018 |
| *Posidonia oceanica* | Po: High fruit dispersal; High clone longevity; Low growth | FE | Gene expression | Nutrients | Phev | Modulation strategy in response to herbivory pressure under nutrient addition | Ruocco et al. 2018 |
| *Posidonia oceanica* | Po: High fruit dispersal; High clone longevity; Low growth | FO | Genotypization; Genome-wide transcriptome analysis | Light and Temperature | L_Adp | Candidate gene for two populations as a signal of natural selection in response to latitudinal and bathymetric gradients. | Jahnke et al. 2019 |
| *Zostera marina* | Zm: High fruit dispersal; High clone longevity; High growth | ME | Chlorophyll a fluorescence; Genotypization; Methylome | Temperature | Phev | Methylation variation in clonal seagrass promotes variation in fitness-related traits | Jueterbock et al. 2019 |
| *Posidonia oceanica* | Po: High fruit dispersal; High clone longevity; Low growth | FE | Leaf morphology; Growth; Shoot abundance | Light | Phev/L_adp | Biogeographical variability related to less genetically diverse populations | Tuya et al. 2019 |
| *Posidonia oceanica* | Po: High fruit dispersal; High clone longevity; Low growth | ME | Photosynthetic performance; shoot growth rate; leaf necrosis | Temperature and Nutrients | Phev | The combined effects of warming and ammonium were much worse than those of each stressor in isolation | Ontoria et al. 2019 |
| *Posidonia australis Zostera muelleri* | Pa: High fruit dispersal; High clone longevity; Low growth  Zm: Low fruit dispersal; Medium-low clone longevity; High growth | CGE | Chlorophyll a fluorescence; Growth; Chlorophylls content; Gene expression; | Temperature | Phev | Intraspecific thermal responses and epigenetic modifications could play a central role in seagrass thermal stress memory. | Nguyen et al. 2020 |
| *Enhalus acoroides* | Ea: High fruit dispersal; High clone longevity; High growth | ME | Morphological Traits; Growth rate; Photosynthetic performance; Nutrient content; Non-Structural carbohydrates; | Temperature and Nutrients (multiple stressors) | Phev | Resilience capacity to temperature and nutrients | Artika et al. 2020 |
| *Thalassia hemprichii;*  *Cymodocea serrulata; Halophila stipulacea* | Te: Medium fruit dispersal; Medium clone longevity; Medium growth  Cs: Low fruit dispersal; Medium-low clone longevity; High growth  Hs: High fruit dispersal; Low clone longevity; High growth | CGE | Nitrogen and carbon content; Non-structural carbohydrate; Morphology; Growth rates; Chlorophyll a fluorescence | Temperature and Nutrients (multiple stressors) | Phev/L_Adp | Different responses and strategies adopted by co-inhabiting seagrasses in face of environmental changes | Viana et al. 2020 |
| *Posidonia oceanica* | Po: High fruit dispersal; High clone longevity; Low growth | CGE | Morphology; Growth rates; Chlorophyll a fluorescence; Chlorophyll content; Nitrogen and carbon content; Non-structural carbohydrate | Temperature and Nutrients (multiple stressors) | Phev/L_Adp | Antagonistic effects of temperature and nutrients and differentiated plants responses according to their native environment | Pazzaglia et al. 2020 |

Note: refer to the main text for complete references
